# Running on empty: Factors underpinning impaired cardiac output reserve in heart failure with preserved ejection fraction

**DOI:** 10.1113/EP091776

**Published:** 2024-02-29

**Authors:** Paula Sagmeister, Sebastian Rosch, Karl Fengler, Karl‐Patrik Kresoja, Tommaso Gori, Holger Thiele, Philipp Lurz, Daniel Burkhoff, Karl‐Philipp Rommel

**Affiliations:** ^1^ Department of Cardiology Heart Center Leipzig at University of Leipzig and Leipzig Heart Science Leipzig Germany; ^2^ Department of Cardiology University Hospital Mainz Mainz Germany; ^3^ Cardiovascular Research Foundation New York New York USA

**Keywords:** cardiac output, haemodynamics, heart failure interventions, heart failure with preserved ejection fraction, pressure–volume‐loops

## Abstract

Heart failure with preserved ejection fraction (HFpEF) is frequently attributed etiologically to an underlying left ventricular (LV) diastolic dysfunction, although its pathophysiology is far more complex and can exhibit significant variations among patients. This review endeavours to systematically unravel the pathophysiological heterogeneity by illustrating diverse mechanisms leading to an impaired cardiac output reserve, a central and prevalent haemodynamic abnormality in HFpEF patients. Drawing on previously published findings from our research group, we propose a pathophysiology‐guided phenotyping based on the presence of: (1) LV diastolic dysfunction, (2) LV systolic pathologies, (3) arterial stiffness, (4) atrial impairment, (5) right ventricular dysfunction, (6) tricuspid valve regurgitation, and (7) chronotopic incompetence. Tailored to each specific phenotype, we explore various potential treatment options such as antifibrotic medication, diuretics, renal denervation and more. Our conclusion underscores the pivotal role of cardiac output reserve as a key haemodynamic abnormality in HFpEF, emphasizing that by phenotyping patients according to its individual pathomechanisms, insights into personalized therapeutic approaches can be gleaned.

## Abstract (highlights)

- **What is the topic of this review?**
  HFpEF represents a complex disease characterized by a heterogeneous pathophysiology. This review offers an overview of key pathophysiological mechanisms contributing to the impairment of cardiac output reserve during exercise in HFpEF, along with potential specific treatment options.
- **What advances does it highlight?**
  To implement a specific treatment option for patients with HFpEF, a thorough understanding of its underlying pathophysiology on an individual basis is crucial. Here, we present the evidence for a novel pragmatic phenotyping based on underlying pathophysiology and discuss its implications for therapeutic strategies.

## 1 INTRODUCTION

Heart failure with preserved ejection fraction (HFpEF) is a commonly diagnosed syndrome with an incidence that is rising with the ageing population and concomitant comorbidities such as arterial hypertension, diabetes mellitus and obesity (Borlaug, 2020). Problematically, sufficient treatment options are very limited. This may be due to significant heterogeneity among patients in this population, with varying contributions of a high burden of comorbidities and reduced aerobic fitness. In the past decade, numerous clinical trials have failed to demonstrate universal benefit for medical therapies in HFpEF. As such, for a long‐time optimal management of comorbidities and exercise training were the only guideline recommendations. It is only recently that a first drug‐based therapy, specifically treatment with a sodium glucose transporter 2 inhibitor, has been shown to significantly reduce HF hospitalizations and improve quality of life in HFpEF (McDonagh et al., 2023). Additional benefits might be derived from medication with mineralocorticoid receptor antagonists or glucagon‐like peptide‐1 receptor agonists in certain subgroups of HFpEF patients (Kosiborod et al., 2023). Overall, the clinical trial experience underlies calls for patient‐specific individualization of treatment.

Heart failure is a state characterized by the heart's inability to supply adequate cardiac output (CO) to the body or to achieve this only at the expense of heightened filling pressures. HFpEF, in particular, is defined as having a normal left ventricular (LV) ejection fraction (EF, with EF ≥50%), as well as evidence of elevated LV filling pressures at rest or during exercise. Most prior studies and reviews focused on the implications and mechanisms of elevated pulmonary capillary wedge pressures (PCWP) and central venous pressures. However, the central haemodynamic abnormalities also prominently include impaired cardiac output reserve (COR) (Del Buono et al., 2019).

Cardiac function hinges on diastolic and systolic physiological processes, both at rest and during exercise, to ensure adequate ventricular filling (preload) to achieve adequate cardiac output. Different impediments to ventricular filling led to an augmentation in PCWP and LV diastolic pressures, potentially resulting in symptoms such as dyspnoea and/or exercise limitation (Borlaug, 2014b; Cheng et al., 1992).

Under normal conditions, several physiological mechanisms contribute to the elevation of CO during exercise (Borlaug, 2014a, 2020; Borlaug & Kass, 2011a). They include an increase in heart rate, systemic and pulmonary arterial vasodilatation, systemic venoconstriction, increased biventricular contractility and biventricular lusitropic reserve facilitating a reduction in ventricular pressures during early diastole and enhancing a ‘suction effect’. The latter allows for increased diastolic ventricular filling despite a shorter diastolic time interval, without causing significant increases in left atrial (LA) and/or right atrial (RA) pressures. Venoconstriction recruits blood from the splanchnic venous reservoir, shifting it to the central circulation to increase preload as signified by the increases in end‐diastolic volume which augments stroke volume (SV) via the Frank–Starling mechanism. As such, inotropy increases, also enhancing elastic recoil, contributing to subsequent improved early diastolic filling. Moreover, ventricular afterload is reduced, which is mediated by a decrease in peripheral vascular resistance (vasodilatation). Increased venous return and pulmonary artery vasodilatation lead to augmented right ventricular (RV) ejection during exercise, ensuring sufficient LV filling without a concomitant increase in pulmonary artery pressure. In summary, under normal healthy conditions, an intricate orchestration of multiple physiological mechanisms ensures that CO increases with only relatively small increases of filling pressures (Hsu et al., 2022).

**FIGURE 1:**
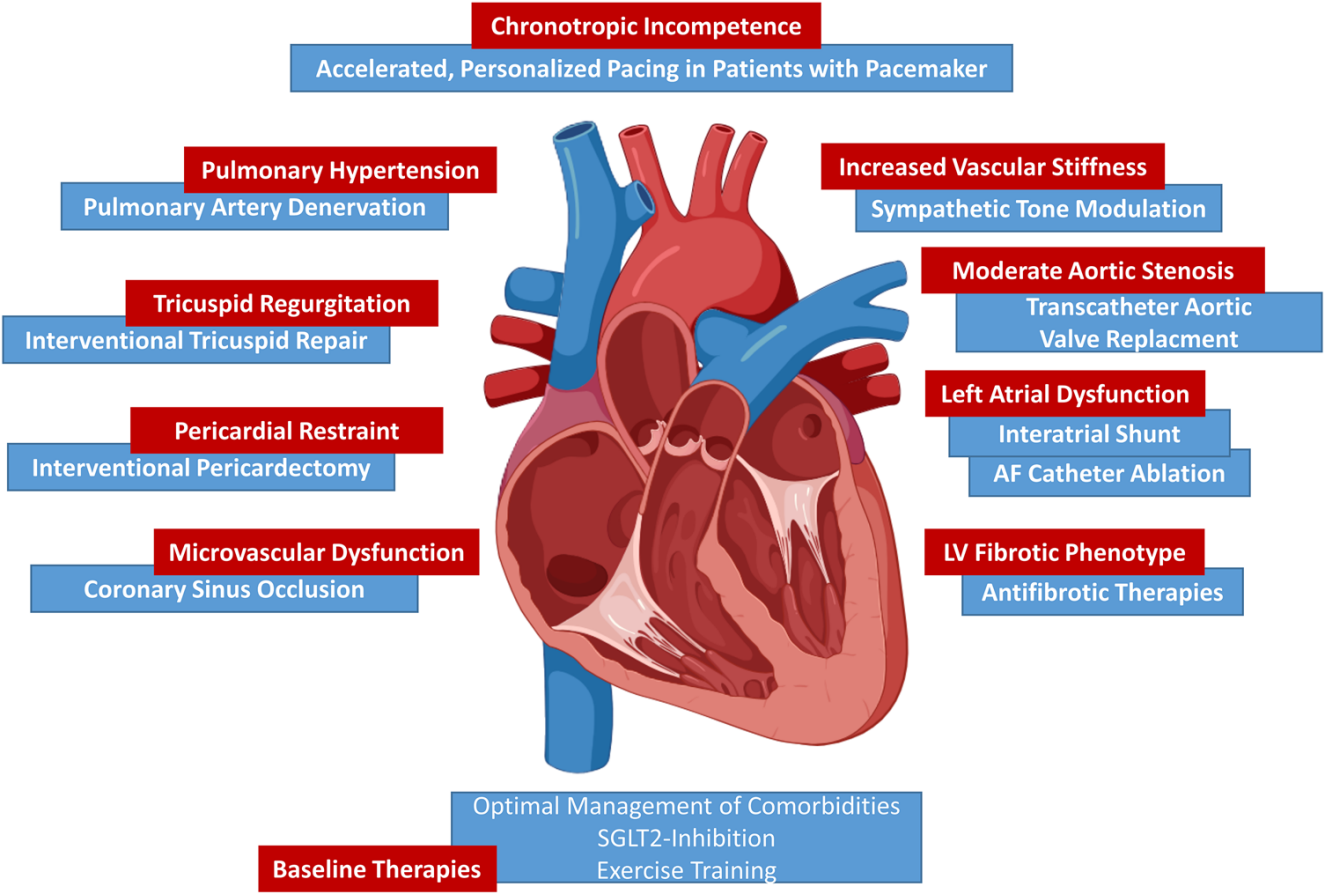
Pathophysiology guided interventional treatment targets to improve cardiac output reserve. This figure exemplarily illustrates targets (red boxes) for interventional therapies to improve cardiac output reserve, which can be identified by pathophysiology‐guided phenotyping in patients with heart failure with preserved ejection fraction (HFpEF). Possible treatment options are highlighted in blue. For details, refer to the discussion section. Independent of the HFpEF phenotype, patients should be treated with a baseline therapy with optimal comorbidity management, a sodium–glucose‐cotransporter (SGLT)2‐inhibitor and exercise training according to the current guidelines (McDonagh et al., 2023). AF, atrial fibrillation; LV, left ventricle.

Individuals with HFpEF typically belong to an older demographic, are more likely to be female and/or obese, and frequently exhibit concurrent conditions such as atrial fibrillation (AF) and/or arterial hypertension. They often present with elevated levels of N‐terminal prohormone of brain natriuretic peptide (NT‐proBNP) and experience exertional dyspnoea. While RV and LV volumes tend to be comparable between HFpEF patients and a healthy population, those with HFpEF exhibit higher systolic and diastolic RV and LV pressures, pulmonary artery pressure, and PCWP. Additional characteristics include prolonged active relaxation of both RV and LV, an upwards‐shifted RV and LV end‐diastolic pressure–volume relationship (EDPVR) during exercise in some patients, inotropic incompetence, insufficient vasodilatation with ventriculo‐arterial uncoupling due to increased arterial afterload, dysregulation of venous tone and chronotopic incompetence (Kaye et al., 2021; Roeder et al., 2017; Rommel et al., 2016, 2018; Sorimachi et al., 2021). These factors collectively impair certain mechanisms, particularly the increase in SV, crucial for achieving an elevation in CO and maintaining a physiological balance between venous return and cardiac output during exercise. This importantly contributes to symptoms such as dyspnoea and/or a diminished exercise capacity. Specific pathologies potentially causing impaired COR during exercise within HFpEF encompass various factors, as depicted in Figure 1 (Borlaug, 2014a; Del Buono et al., 2019; Obokata et al., 2020). These include LV diastolic dysfunction with inadequate ventricular filling and activation of the Frank–Starling mechanism, ventricular systolic dysfunction, vascular stiffening leading to ventriculo‐arterial uncoupling, LA dysfunction, RV and/or RA dysfunction, pulmonary hypertension (PH), impaired LV filling through tricuspid valve regurgitation (TR) (Reddy et al., 2023), and chronotropic incompetence.

This review aims to explore diverse pathophysiologies of HFpEF, examining their impact on the compromised cardiac output during exercise and potential therapeutic targets

## 2 ALTERATIONS IN LEFT VENTRICULAR STRUCTURE AND FUNCTION

In the majority of HFpEF patients, there is evident structural remodelling of the LV characterized by a concentric pattern and histological changes in cardiomyocytes and the extracellular matrix. This remodelling is believed to significantly contribute to increased LV stiffness and underfilling. It is important to note, however, that the absence of LV remodelling does not necessarily exclude HFpEF (van Heerebeek et al., 2006). While diastolic dysfunction of the LV plays a pivotal role in HFpEF, it should not be used synonymously, as diastolic dysfunction can also be present due to ageing and comorbidities such as arterial hypertension or diabetes mellitus in asymptomatic patients without HFpEF.

LV diastolic dysfunction encompasses various aspects, including impaired active myocardial relaxation with an additional inability to augment relaxation during exertion, increased end‐diastolic LV stiffness, and decreased distensibility of the LV, along with elevated end‐diastolic LV (and LA) pressures at rest and/or during exercise (Zile et al., 2004). The compromised LV myocardial relaxation leads to impairments in early LV filling and consequently an increased contribution of the active LA contraction during late diastole to maintain proper LV filling. However, during exertion, with an increase in heart rate and subsequent shortening of late diastole, this compensatory mechanism is disrupted, resulting in further underfilling of the LV (Borlaug, 2014a; Wachter et al., 2009). Active relaxation is an energy‐dependent process and one of the earliest functional alterations in the context of reduced myocardial energy supply. In HFpEF, this reduction may occur for various reasons, including microvascular dysfunction and significant changes in myocardial uptake and utilization of energy substrates (Capone et al., 2023).

**FIGURE 2:**
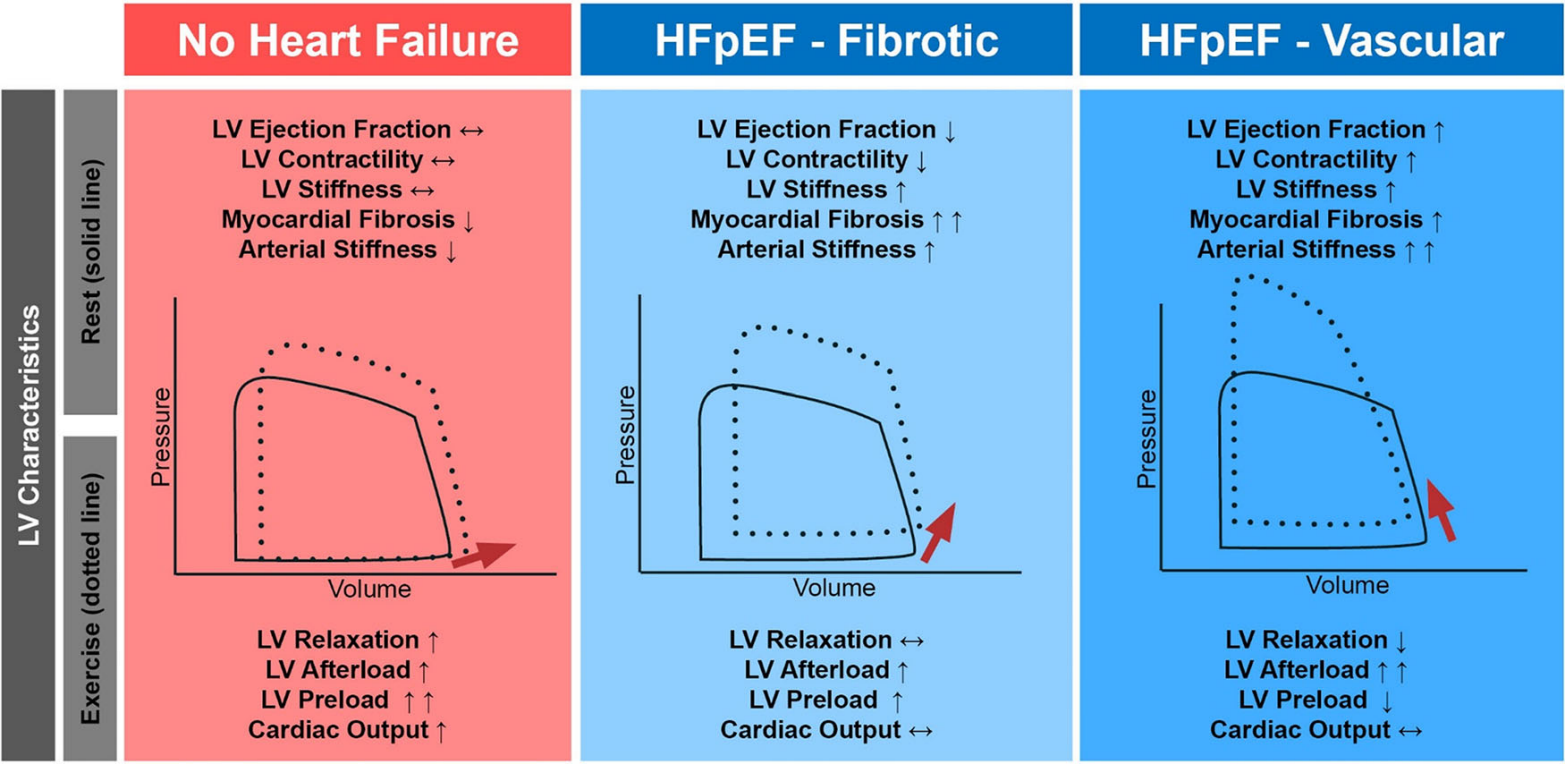
Phenotyping according to LV structure and function. Schematic illustration, based on prior studies Rommel et al. (2016) and Rosch et al. (2022), depicting mechanisms of impaired left ventricular (LV) filling and subsequent reduction in cardiac output reserve in patients with heart failure and preserved ejection fraction (HFpEF), along with the associated phenotypic heterogeneity. In individuals without heart failure, the pressure–volume‐loop shifts to the right along a shallow slope of the end‐diastolic pressure–volume relationship, resulting in minimal increases in end‐diastolic pressures (red panel, red arrow). Conversely, patients with HFpEF experience higher elevations in both end‐diastolic and aortic pressures. For patients with LV ejection fraction in the lower ranges of normal and diffuse myocardial fibrosis, a steep slope is observed in their end‐diastolic pressure–volume relationship, leading to exaggerated elevations in end‐diastolic pressures with any given increase in LV preload (light blue panel, note the arrow pointing right and up). However, in patients with relatively high left ventricular ejection fractions, and heightened chamber stiffness despite limited myocardial fibrosis, as well as an elevated vascular stiffness, a paradoxical shift of the pressure–volume loop upwards and to the left is observed. This implies a change of the end‐diastolic pressure–volume relationship with a reduction in volumetric LV filling and preload despite excessively high end‐diastolic and aortic pressures (dark blue panel, note that the arrow points to the left).

A study by our group investigating myocardial fibrosis in HFpEF patients measured by extracellular volume fraction in cardiac magnetic resonance revealed significantly higher extracellular volume fraction in HFpEF patients compared to those without heart failure. This was associated with higher LV end‐diastolic pressure at baseline and during exercise, prolonged active relaxation during exercise, and a significantly higher load‐independent LV stiffness constant. These factors collectively led to a more pronounced upward shift in the LV EDPVR during exertion. Interestingly, markers of systolic function did not differ. These findings suggest a HFpEF phenotype with higher extracellular volume fraction/interstitial myocardial fibrosis associated with increased stiffness and impaired filling. However, a subgroup of patients with HFpEF characterized by low extracellular volume fraction and less pronounced LV diastolic stiffness was identified, showing similarly impaired filling under exercise due to increased vascular load on the LV, impaired ventriculo‐arterial coupling and impairments in active relaxation. Importantly, LV preload recruitment seemed to be impaired in these patients, as invasively acquired pressure–volume loops under exertion (and thus the EDPVR) shifted upwards instead of to the right, as depicted in Figure 2 (Rommel et al., 2016).

Another classification of HFpEF was proposed according to LV‐EF. HFpEF patients with low‐normal LV‐EF (EF 50–60%) were characterized by more myocardial fibrosis, larger LV chambers, lower intrinsic LV contractility and impaired ventriculo‐arterial coupling. In contrast, HFpEF patients with supra‐normal LV‐EF (EF ≥60%) had higher chamber stiffness in diastole and systole despite having less myocardial fibrosis, increased LV afterload and diminished preload reserve.

The reasons for these observations remain speculative, but the study highlights the crucial role of systolic LV (dys)function and systolic–diastolic interaction in patients with HFpEF, despite the definition of a preserved LV‐EF (Rosch et al., 2022). During handgrip exercise, the pressure–volume loops shifted to the right along the EDPVR in the cohort with EF 50–60%, while a EDPVR shift upwards with higher LV end‐diastolic and LV end‐systolic pressure was observed in the cohort with EF ≥60% (Figure 2). The reasons for these observations remain speculative, but include a pathological interventricular interaction with a contribution of pericardial restraint to impaired ventricular filling, and impaired energy‐dependent active myocardial relaxation (Borlaug & Reddy, 2019; Brener et al., 2022). The study highlights two important points: (1) ventricular chamber stiffness is not only determined by myocardial fibrosis but also by other factors like cellular stiffness and chamber geometry, and (2) LV dysfunction might be present despite evidence of a preserved LV‐EF in HFpEF.

## 3 LARGE ARTERY STIFFNESS AND LEFT VENTRICULAR AFTERLOAD

While large artery stiffness has been suggested as a potential contributor to HFpEF pathophysiology, it is not always straightforward to discern physiological vascular changes associated with ageing from those associated with pathology (Chirinos et al., 2019). However, a distinct HFpEF phenotype appears to be linked with increased afterload due to arterial hypertension, increased vascular stiffness (with associated increased pulsatile load), and abnormally high sympathetic nervous activity (Chirinos et al., 2019). These patients exhibited unfavourable haemodynamics with low aortic distensibility, high blood pressure variability, high SV index, and increased myocardial work at rest (Borlaug & Kass, 2011b; Chirinos et al., 2019; Kresoja, Rommel, Fengler, et al., 2021). As a compensatory mechanism invoked to maintain favourable ventriculo‐arterial coupling, LV contractility increases to increase CO, but the decreased vascular compliance results in marked and disproportionate increases of blood pressure during exercise which contributes to abnormally increased filling pressure and, ultimately, poor exercise intolerance. Therefore, improving arterial compliance might be an attractive target in this subgroup of HFpEF patients which may also impact on cardiac remodelling and dysfunction.

**FIGURE 3:**
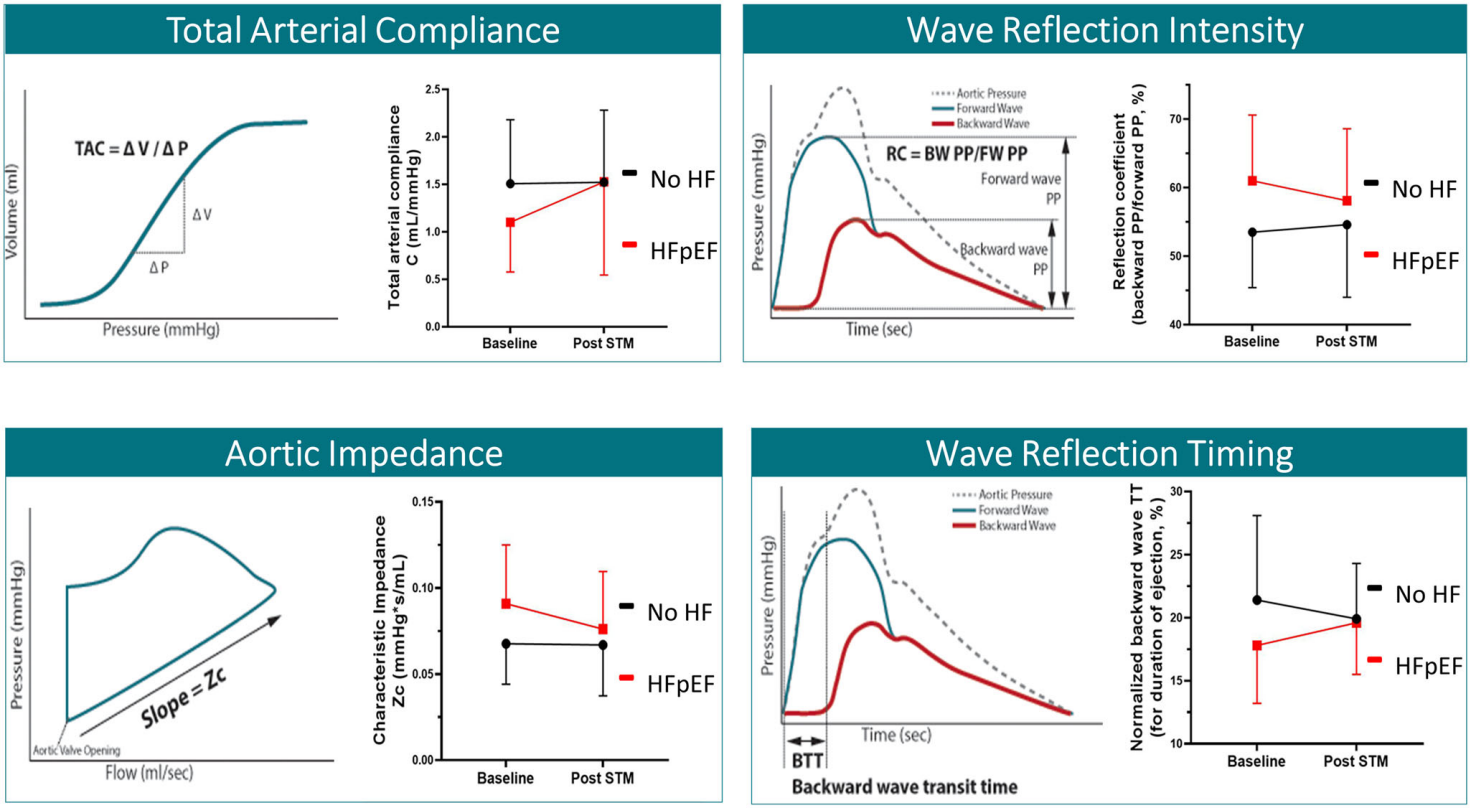
Large artery stiffness. This figure, modified from Rommel et al. (2023), displays the effects of sympathetic tone modulation (STM) on large artery stiffness and function. Aortic stiffness leads to various haemodynamic alterations of aortic function: (1) decrease in total arterial compliance (TAC); (2) increased characterized impedance of the proximal aorta (Zc); (3) augmentation of the reflected pulse wave (reflection coefficient (RC) = backward wave (BW) pulse pressure (PP) / foward wave (FW) PP); and (4) premature (late‐systolic) arrival of the reflected wave, as evident by a shorter backward wave transit time (BTT). STM by interventional renal denervation attenuated pathological arterial function in HFpEF.

Renal denervation (RDN) has been established as a blood pressure‐lowering therapy achieved through interventional attenuation of sympathetic activity, accomplished by ablating efferent and afferent sympathetic nerves along the renal arteries. The procedure is deemed safe and effective, thus making it well‐positioned to address the highly prevalent comorbidity of arterial hypertension in patients with HFpEF. Furthermore, following RDN in patients with uncontrolled arterial hypertension, irrespective of concomitant HFpEF, improvements in aortic distensibility, normalization of SV index and CO index have been observed (Kresoja, Rommel, Fengler, et al., 2021; Rommel et al., 2023). In addition, RDN is associated with a reduction in pathologically increased arterial stiffness in hypertensive patients with HFpEF. A study by Kresoja et al. showed that RDN partially normalized haemodynamics in the subgroup of patients with HFpEF. Reduced pulsatile load presumably mediated by improvement of aortic distensibility and an associated reduction of *E*/*e′* (a surrogate of LV filling pressure) were observed. Correspondingly, NT‐proBNP levels decreased significantly, and over 20% of patients with HFpEF undergoing RDN showed an improvement of at least one class of the New York Heart Association classification (Kresoja, Rommel, Fengler, et al., 2021). In addition, attenuation of a hypercontractile state was observed in patients with supra‐normal LV‐EF (i.e., EF > 60%) in response to RDN (Fengler et al., 2022). Recently, we investigated pulsatile LV loading using a mathematical haemodynamic model. We observed that patients with HFpEF displayed elevated pulse wave velocity and pulse pressure, reduced total arterial compliance, higher characteristic impedance of the proximal aorta, as well as premature wave reflections, with high wave reflection magnitudes, indicating an impaired ventricular–arterial coupling. In simpler terms, a high arterial stiffness leads to early arrival of wave reflections and higher impedance to LV ejection which, in turn, causes increases in LV wall stress. Reassessing arterial function 3 months after RDN showed that these arterial properties were partly normalized in HFpEF patients, indicating improvement and partial normalization of large artery stiffness and ventricular–arterial coupling. Importantly, there was no significant change in arterial properties in the control cohort after RDN (Figure 3) (Chirinos et al., 2019; Rommel et al., 2023).

Moreover, it has been proposed that the attenuation of sympathetically mediated peripheral vasoconstriction during exercise, a concept called functional sympatholysis, is impaired in patients with HFpEF, further underlining the importance of sympathetic activity for vascular function (Alpenglow et al., 2023).

Similar to the stiffness observed in large arteries, the existence of aortic stenosis amplifies LV afterload. A noteworthy prevalence of low‐grade aortic stenosis, exceeding 50%, has been observed in patients hospitalized for HFpEF. Haemodynamic alterations in HFpEF patients with low‐grade aortic stenosis, compared to those without, encompass heightened LV contractility, increased SV, and smaller, less distensible LVs, paralleling the characteristics of patients with large artery stiffness, as discussed earlier (Verbrugge et al., 2021). More recently, a study has proposed that, within a cohort of patients experiencing moderate to severe aortic stenosis, deficiencies in exercise capacity and exercise haemodynamics are dictated more by the presence of HFpEF rather than the severity of stenosis. Notably, in patients with HFpEF and aortic stenosis, an impaired COR was identified as a significant contributor to cardiac limitations (Hoedemakers et al., 2023).

## 4 ATRIAL FUNCTION MODULATES VENTRICULAR FILLING

Numerous HFpEF‐diagnosed patients exhibit enlarged LAs due to remodelling and dysfunction, commonly associated with AF. Evidence indicates that compromised LA function is linked to an unfavourable prognosis and an elevated incidence of PH in patients with HFpEF, even in the absence of AF. This underscores the importance of comprehending and addressing LA impairments in the context of HFpEF (Borlaug, 2014b; Weerts et al., 2021).

Physiologically, the LA is passively filled with blood from the pulmonary veins during LV contraction through a suction mechanism (reservoir function). In early diastole, blood is passively drawn into the LV (conduit function), and actively pushed into the the LV by means of atrial contraction during late diastole (active booster pump function) (Kono et al., 1992; Marino, 2021).

As compared to individuals without heart failure, patients with HFpEF display reduced LA function as indicated by reductions in volumetric or stain derived indices, specifically lower total LA EF, reservoir stain and conduit strain. Interestingly, active LA booster pump function initially remains preserved. Compensated by LA dilatation and increased contraction, LA stroke volume is even slightly elevated in HFpEF patients at rest. However, as the disease progresses or during exercise, passive LA function and specifically conduit function further diminish until this compensatory mechanism ultimately fails, leading to inadequate early ventricular filling, impaired COR and exercise intolerance (Roeder et al., 2017). The dependence of LV filling on active LA booster pump function during the course of HFpEF explains the detrimental impact of paroxysms of AF on clinical status of these patients. Besides a progressive and prognostically relevant decrease in reservoir strain over time, the occurrence of AF ultimately leads to a loss of active LA contraction and therefore further impairs LV filling and COR (Roeder et al., 2022, 2020). As such, a progressive LA cardiomyopathy has been suggested to be an independent and progressive pathomechanistic entity in patients with HFpEF (Reddy et al., 2020).

In fact, elevated left atrial pressures or PCWP are associated with adverse left atrial remodelling, impaired exercise capacity, and poor long‐term outcomes. An iatrogenic, pressure‐dependent, left‐to‐right atrial shunt has the potential to decompress the pressure‐overloaded left atrium. This decompression may subsequently counteract adverse left atrial remodelling, improve exercise capacity, and decrease heart failure‐related poor outcomes (Salah et al., 2024).

Similarly, RA reservoir and conduit function are reduced, and RA booster pump function is compensatorily increased in patients with early HFpEF at rest. However, during exercise, an increase in heart rate shortens diastole, impeding the contribution of active RA contraction, resulting in RV underfilling. Of note, some authors attribute structural and functional changes in the right side of the heart, particularly in the RA, to be secondary to left‐sided changes, or at least occurring later in the temporal progression of diseases. Therefore, the concept of an independent RA cardiomyopathy is less established (Borlaug, Sharma, et al., 2023).

## 5 RIGHT VENTRICULAR DYSFUNCTION AND PULMONARY HYPERTENSION

Similar to LV pathologies, RV diastolic and systolic dysfunction, present in approximately 20–35% of HFpEF patients, play a crucial role in the pathophysiology of HFpEF and contribute to the impairment of CO during exercise. Patients with a lower LV‐EF and/or concomitant AF are more susceptible to the development of systolic RV dysfunction and severe PH. Independent of the severity of PH, the existence of RV dysfunction serves as a significant risk factor for an increased morbidity and mortality in HFpEF (Melenovsky et al., 2014; Reddy & Borlaug, 2021; Sanz et al., 2019). In a longitudinal study, 23% of patients with HFpEF and initially normal RV function developed RV dysfunction over a median follow‐up period of 4 years [Borlaug et al., 2023].

We could show that even in early stages of HFpEF without overt systolic RV dysfunction, evidence of RV diastolic dysfunction can be apparent, contributing to impaired LV filling and COR. While RV‐EF, RV end‐systolic and RV end‐diastolic volume, RV‐SV and thus RV‐CO at rest were similar in HFpEF patients and patients without heart failure, systolic and diastolic RV pressures were elevated at rest in the HFpEF population. The RV‐pulmonary coupling ratio was lower in HFpEF patients, indicating a systolic overcompensation. Physiologically, one can expect an increase in RV end‐diastolic and RV end‐systolic pressure, RV end‐diastolic volume and PCWP, as well as heart rate during exertion to cover the increase in oxygen demand through an augmentation in CO. While some of these adaptations were observed in our study, the rise in RV end‐diastolic pressure and PCWP were far greater, and the rise in heart rate less distinct in patients with HFpEF when compared to patients without heart failure (Rommel et al., 2018).

**FIGURE 4:**
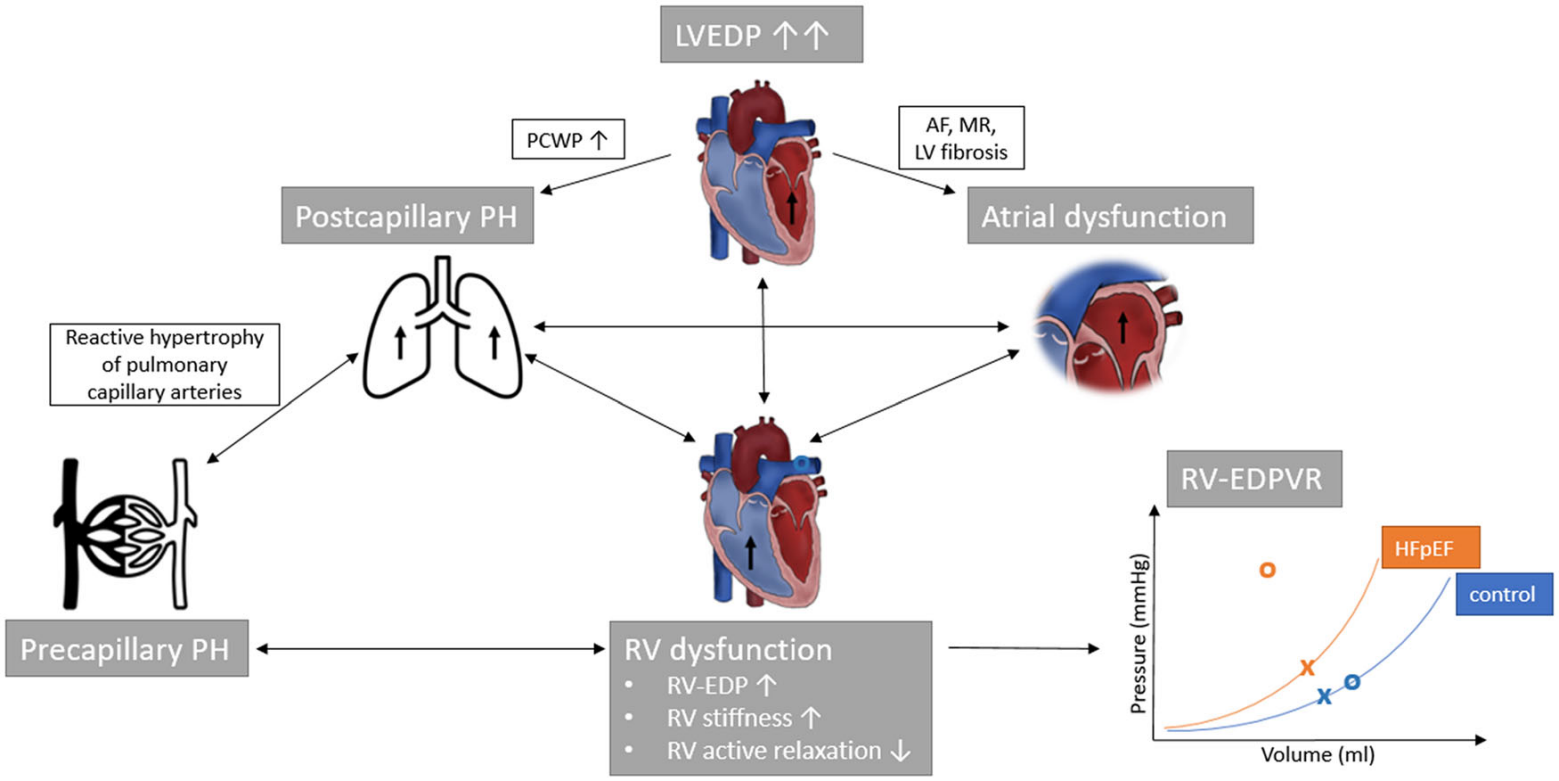
RV dysfunction. Modified from Kresoja et al. (2023) and Rommel et al. (2018), this schematic illustration displays the pathophysiology of right ventricular (RV) dysfunction in heart failure with preserved ejection fraction (HFpEF). Factors affecting structural myocardial changes affect both RV and left ventricle (LV). In addition, increase of LV end‐diastolic pressure (EDP) promotes increase in pulmonary capillary wedge pressure (PCWP) as well as atrial dysfunction. Atrial dysfunction is further associated with atrial fibrillation (AF), mitral valve regurgitation (MR) and atrial fibrosis. These factors lead to the development of pulmonary hypertension (PH), with subsequent structural changes of the pulmonary vasculature, increasing RV afterload and wall stress. As such PH promotes and further exacerbates RV dysfunction. RV‐EDP and RV stiffness rise and HFpEF patients exhibit a steeper slope of the RV end‐diastolic pressure–volume relationship (RV‐EDPVR). In addition, under exercise RV pressure–volume loops shift upwards in some patients (orange curve; × symbolizes EDPVR at rest and the circle at exercise) as compared to the physiological shift to the right along the EDPVR in the control cohort (blue curve).

Due to the combination of an increased load‐independent RV stiffness and an impaired RV active relaxation, the expected increase in RV end‐diastolic volume during exertion was absent in patients with HFpEF, leading to an inadequate increase of RV filling, a decrease in SV, and consequently an inability to augment CO during exertion. Consequently, the RV‐EDPVR shifted upwards, increasing end‐diastolic pressure without an accompanying increase in end‐diastolic volume. This paradoxical EDPVR upwards shift with markedly elevated ventricular filling pressures mirrors the observations made for the LV and reflects an inability to adequately enhance preload and consequently CO. Hence, the RV displays similar diastolic abnormalities as the LV in patients with HFpEF, and structural and functional changes of the RV should be considered as independent pathophysiological factors (Figure 4) (Rommel et al., 2018).

The pathognomonically increased LV filling pressures and LA pressures lead to increased pulmonary artery pressures via a backward transmission, a phenomenon referred to as postcapillary PH (Rommel et al., 2018). However, in more advanced stages, a precapillary component can develop due to alterations in pulmonary vascular structure and function (Reddy & Borlaug, 2021). In general, 60–75% of the HFpEF population exhibits PH which is associated with increased risks of mortality and hospitalization.

## 6 TRICUSPID REGURGITATION AND PERICARDIAL RESTRAINT AS CAUSE OF LEFT VENTRICULAR UNDERFILLING

The high prevalence and important implications of advanced stages of TR are being increasingly recognized. In the majority of cases, TR is attributable to an underlying condition, making it of secondary or functional origin. Recently, further subclassification has been proposed into atrial‐functional TR, typically associated with RA enlargement and long‐standing AF; and ventricular functional TR, which arises due to RV remodelling as a consequence of PH, cardiomyopathies involving the RV, RV ischaemia, or arrhythmias (Hahn et al., 2023). The occurrence of TR is frequent among patients with HFpEF, and evidence of advanced isolated TR in the presence of a normal LV‐EF should always prompt suspicion for HFpEF (Hahn, 2023; Hsu et al., 2022; Santas et al., 2015).

**FIGURE 5:**
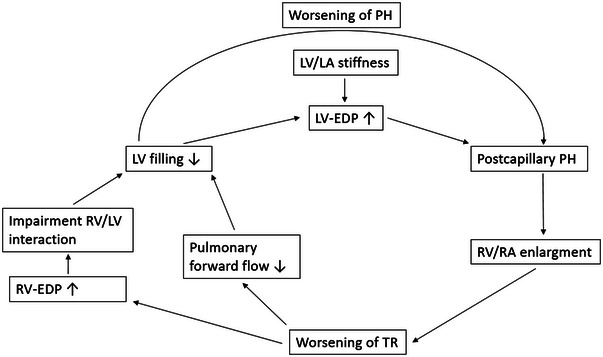
Tricsupid regurgitation and HFpEF. This figure displays the vicious cycle in patients with heart failure with preserved ejection fraction (HFpEF) and concomitant tricuspid valve regurgitation (TR) as adapted from Rommel et al. (2019) and Kresoja et al. (2023). Left ventricular (LV) pathologies lead to an increase in LV end‐diastolic pressure (EDP) and pulmonary capillary wedge pressure (PCWP), which can result in a right ventricular (RV) and/or right atrial (RA) enlargement. This initiates and consequently exacerbates TR. Due to high RV regurgitation volume and a resulting decrease in pulmonary forward flow, an underfilling of the LV occurs. Similarly, elevated RV volumes and RV‐EDP cause a deviation of the septum to the left, causing unfavourable RV/LV interaction and ultimately LV underfilling. With progression of this mechanism, LV filling pressure rises further and PH worsens, completing the cycle in the HFpEF phenotype with concomitant TR.

From a haemodynamic perspective, TR plays a significant role in compromising COR by contributing to RV regurgitation volume, diminishing RV forward SV, decreasing LV filling volume, and impeding LV filling by means of increasing LV filling pressures through enhanced ventricular interdependence (Figure 5) (Andersen et al., 2014; Kresoja et al., 2023; Rommel et al., 2019). Ventricular interdependence describes the impact of loading and function of one chamber on the other. In patients with TR, chronic RV volume overload and increased RV end‐diastolic pressure, there is a leftward shift of the septum during diastole, resulting in impaired LV filling in the restricted pericardial space (Lurz et al., 2009). Additionally, reduced pulmonary forward flow contributes to reduced LV preload and, according to the Frank–Starling mechanism, contributes to decreased LV‐SV (Andersen et al., 2014). Therefore, patients with HFpEF and concomitant TR typically have reduced CO despite potentially normal LV function. In order to maintain normal RV‐SV, compensatory RV dilatation occurs, ultimately aggravating RV–LV interaction, and leading to further impairments of LV filling (Rommel et al., 2019). Recently, interventional approaches have been suggested to reduce TR. Besides their potential to alter the clinical trajectory of these patients, they can also serve as a physiological model. We investigated the haemodynamic changes following transcatheter edge‐to‐edge repair (TEER) in patients with severe TR. Although this study was not specifically designed for patients with HFpEF, 45% of patients in this study were concomitantly diagnosed with HFpEF. After successful TEER, cardiac magnetic resonance findings revealed a decrease in RV volume overload, and hence a decrease in RV end‐diastolic volume and total RV‐SV, accompanied by an increase in effective pulmonary forward flow. This led to improvements in LV filling, resulting in an increase in LV end‐diastolic volume and LV‐SV, likely due to relief of interventricular interactions. The rise in LV‐SV led to an augmentation of CO and improvement of exercise performance as evidenced by a 20% increase in the 6‐min walk distance at 1 month after TEER. Interestingly, an increase in *E*/*E′* following TEER was apparent, consistent with increased LV filling, but raising the question of whether the improvement of TR could potentially worsen HFpEF symptoms (Rommel et al., 2019).

However, investigating HFpEF patients undergoing TEER for TR with the invasive assessments of pressure–volume loops demonstrated a stable LV end‐diastolic pressure despite increased LV end‐diastolic volume after TEER. This can only be explained by a downward and rightward shift of the EDPVR, indeed implying that LV filling was improved by means of the reduction in ventricular interdependence. Therefore, interventional treatment options for TR might be associated with better outcomes in this particular subset of patients with HFpEF and severe TR and might not only lead to an improvement in TR but also HFpEF‐specific alterations (Kresoja et al., 2023).

Importantly, other HFpEF phenotypes have been described to exhibit a pathological interventricular interaction, such as the ‘obese HFpEF’ phenotype (Obokata et al., 2017). A special role here is attributed to the epicardial fat, which might not only exert biochemical but also mechanical effects. As such, increased epicardial fat volumes are associated with insulin resistance, systemic inflammation and increases in myocyte injury. Moreover, they are linked to more profound haemodynamic derangements at rest and during exercise, including greater elevation in cardiac filling pressures, more severe pulmonary hypertension and greater pericardial restraint. These factors culminate in poorer exercise capacity (Koepp et al., 2020). Additionally, adipose tissue expansion is associated with adipocyte dysfunction, potentially exerting endocrine and paracrine effects on the myocardium (Kresoja, Rommel, Wachter, et al., 2021).

## 7 CHRONOTROPIC INCOMPETENCE

In healthy subjects heart rate increases approximately 2.5‐fold, while SV increases about 1.5‐fold during peak exercise, emphasizing the important role of heart rate augmentation for COR. Chronotropic incompetence, defined as the inability to adequately augment heart rate during exercise, is observed in over 55% of HFpEF patients. Coupled with the known limitation in SV reserve in HFpEF, chronotropic incompetence significantly contributes to the inadequate COR (Brubaker & Kitzman, 2011). Mechanistically, there is a suggestion that cardiac β‐receptor sensitivity may be impaired in some patients with HFpEF. This sets the stage for a critical evaluation of the use of medical beta‐blockade in these patients (Palau et al., 2021; Sarma et al., 2020). Additionally, other mechanisms might contribute to chronotropic incompetence. Baroreflex activation, for instance, increases cardiac output via an increase in heart rate in response to relative hypotension. In turn, chronic hypertension or atherosclerosis (highly prevalent in patients with HFpEF) can reset or attenuate baroreflex activity, leading to sympathetic overactivation despite inadequate increases in heart rate (Ogoh et al., 2003; Raven et al., 2006). Intriguingly, chronotropic incompetence has been proposed to be present even in situations involving healthy individuals, potentially paralleling frequent conditions seen in patients with HFpEF, such as anaemia or hypoxia. While the exact reasons for these observations and the pathophysiological transferability to HFpEF remain elusive, they illustrate the complex mechanisms of cardiac output reserve (COR) control in both health and disease (Stöhr, 2022).

## 8 DISCUSSION

One of the central haemodynamic abnormalities in HFpEF is impaired COR. While chronotropic incompetence and impaired increase in SV during exercise can be seen as the main contributors, detailed investigations to assess the contributions of the multitude of underlying mechanisms is necessary to understand the complex pathophysiology of HFpEF and determine potential treatment targets.

A key contributor to impaired COR is an impaired SV increase during exertion. Among the mechanisms are LV pathologies, increased arterial stiffness and LV afterload, atrial dysfunction, RV dysfunction, PH, and TR. Importantly, potential therapies tailored to specific pathophysiological mechanisms impairing COR in patients with HFpEF have emerged in preliminary investigations (Figure 1).

Some patients exhibit a fibrotic HFpEF subtype with higher extracellular volume fraction, associated with increased LV stiffness, high LV end‐diastolic pressure and prolonged active relaxation time during exercise. Following the approval of pirfenidone, an oral agent with anti‐fibrotic and anti‐inflammatory characteristics, for the treatment of idiopathic pulmonary fibrosis (King et al., 2014), the PIROUETTE trial was conducted. This randomized, placebo‐controlled phase II trial in patients with HFpEF and myocardial fibrosis, defined as extracellular volume fraction ≥27%, showed that pirfenidone reduced myocardial extracellular volume fraction (Lewis et al., 2021). While this hints at a very promising therapeutic option for a fibrotic HFpEF phenotype, phase III trials with a larger population group are required to confirm the effect of pirfenidone in patients with HFpEF before drawing further conclusions.

Another specific HFpEF phenotype is characterized by increased vascular stiffness and pathogenic heightened pulsatile LV afterload. It is important to highlight that these patients are also characterized by an increased LV chamber stiffness despite lower levels of myoacardial fibrosis, illustrating the role of alternative reasons for a reduced LV distensibility, such as cellular stiffness or chamber geometry. In this specific HFpEF phenotype, sympathetic tone modulation by RDN has been suggested to partially normalize haemodynamic alterations and convey clinical benefit (Kresoja, Rommel, Fengler, et al., 2021; Rommel et al., 2023). A prospective, randomized trial (registration: URL: https://www.clinicaltrials.gov; unique identifier: NCT05030987) is currently underway to enhance our understanding of neuromodulation by RDN in HFpEF. In addition, other forms of neuromodulation have been proposed as a therapeutic option in HFpEF. Splanchnic denervation is thought to attenuate pathological venous constriction and impaired vasodilatation (Fudim et al., 2022). Baroreflex activation therapy reduces sympathetic activity and improves exercise capacity as well as quality of life in HFrEF patients (Gronda et al., 2014; Zile et al., 2020). Whether these observations can be transferred to a broader HFpEF population will need to be demonstrated.

Low‐grade aortic stenosis also increases LV afterload and is associated with unfavourable haemodynamics, limited exercise capacity and adverse outcomes in patients with markers of cardiac damage and/or HFpEF (Hoedemakers et al., 2023; Ludwig et al., 2021; Verbrugge et al., 2021). In addition, transcatheter aortic valve replacement improves diastolic function in patients with aortic stenosis, which is associated with favourable survival (Ong et al., 2020). As such, it might be reasonable to assume that transcatheter aortic valve replacement might have the potential to provide clinical benefit in HFpEF patients, which will need to be scrutinized in large ongoing randomized trials (registration: URL: https://www.clinicaltrials.gov; unique identifier: NCT04889872 and NCT05149755).

Atrial dysfunction has been targeted interventionally with the implantation of an intra‐atrial shunt device, aimed at reducing prognostically and functionally relevant elevations in PCWP during exercise in a HFpEF phenotype characterized by a left atrial hypertension in the presence of low RA pressures (Feldman et al., 2018). The REDUCE‐LAP‐HF II trial, however, failed to meet its primary endpoint, which was a composite of cardiovascular death, total heart failure events and health status in patients with intra‐atrial shunt device versus sham control (Shah et al., 2022). Nevertheless, multiple studies are underway testing this therapeutic concept in patients with HFpEF (e.g., registration: URL: https://www.clinicaltrials.gov; unique identifier: NCT05425459 or NCT03499236 or NCT05686317). Rhythm control in patients with heart failure and AF might not only preserve active LA booster pump function, but also attenuate the decline in passive LA function and LA remodelling. Catheter ablation might be appealing to reduce AF burden; however, it might further reduce LA compliance through scar formation (Reddy et al., 2020). While some haemodynamic benefits have been suggested for this interventional approach in a small mechanistic study (Chieng et al., 2023), ongoing trials will have to establish its efficacy in broader HFpEF populations (e.g., registration: URL: https://www.clinicaltrials.gov; unique identifier: NCT05508256).

Recently, the TRILUMINATE trial investigated the effect of the TEER procedure in patients with severe TR. It demonstrated great safety and efficacy in reducing TR, associated with marked improvements in quality of life. However, no significant reduction in either all‐cause mortality or heart failure hospitalization after TEER was noted (Sorajja et al., 2023). Different lines of argumentation have been brought forward to explain these findings, mainly low event rates and early‐stage TR. However, it might well be that the lack of invasive evidence of HFpEF in many patients might have attenuated effects on hard clinical endpoints, as suggested by observational data (Kresoja et al., 2023; Rommel et al., 2019). Hence, we believe that specifically in TR associated with HFpEF, interventional strategies that lead to a reduction in TR can also result in an improvement of HFpEF‐specific alterations. Results of rigorously designed, ongoing trials are highly anticipated to further discern TR treatment effects among patients with different types of heart failure (e.g., registration: URL: https://www.clinicaltrials.gov; unique identifier: NCT04634266 or NCT04782908).

Pericardial restraint has also been suggested as a contributor to adverse haemodynamics and a reduced COR in the ‘obese HFpEF’ phenotype. Pericardiotomy has consequently been shown in animal models to reduce exercise‐induced PCWP elevation, and improve LV filling and COR (Borlaug & Reddy, 2019). In a first dedicated clinical study using a surgical approach in four HFpEF patients the intervention seemed to improve PCWP rise with leg raise and improve quality of life. However, adverse events were frequent and it will need to be demonstrated whether the favourable signals observed in this pilot study can outweigh the potential risks in future controlled trials in larger patient populations using less invasive, percutaneous pericardiotomy devices (Borlaug, Schaff, et al., 2023).

Many patients with HFpEF have concomitant PH, commonly associated with TR and RV dysfunction. While no therapies targeting PH specifically have shown a significant benefit in HFpEF patients, the use of diuretics leading to a reduction of LV and LA pressures was shown to decrease heart failure hospitalization rates (Adamson et al., 2014). Pulmonary artery ablation has emerged as an investigational approach to lower pulmonary artery pressures and resistance in patients with heart failure and combined pre‐ and post‐capillary PH, with promising effects on COR, exercise capacity, cardiac function and clinical outcomes (Zhang et al., 2023). Multiple studies are ongoing to establish the haemodynamic benefits of this approach in HFpEF patients and determine optimal candidates for the procedure (e.g., Registration: URL: https://www.clinicaltrials.gov; unique identifier: NCT06052072).

Chronotropic incompetence is another key contributor to impaired COR and can potentially be addressed by cardiac pacing. Indeed, recent evidence has demonstrated that in patients with HFpEF and an already implanted pacemaker, treatment with a moderately accelerated, personalized pacing rate was safe and improved quality of life, NT‐proBNP levels, physical activity and AF compared to conventional settings (Infeld et al., 2023). However, the RAPID‐HF trial showed no benefits in exercise haemodynamics, and specifically no improvement in COR, or exercise capacity in HFpEF patients implanted with a rate‐responsive pacemaker to specifically increase heart rate during exercise (Reddy et al., 2023). Whether, personalized pacing rate exerts beneficial effects in a more general HFpEF population without pre‐existing pacemakers will have to be demonstrated in ongoing trials in the field (e.g., registration: URL: https://www.clinicaltrials.gov; unique identifier: NCT05839730).

In addition, HFpEF is known to be associated with various comorbidities, such as diabetes mellitus, obesity and coronary artery disease. Indeed, optimal treatment of these comorbidities is, besides the medical sodium glucose transporter 2‐inhibition, one of the few guideline‐recommended therapeutic options in many patients, including optimal coronary revascularization (Hwang et al., 2014; McDonagh et al., 2023). Microvascular dysfunction is a comorbidity with a high prevalence among HFpEF patients, as it might be a common final pathway of different cardio‐metabolic disturbances thought to elicit cardiac alterations (Shah et al., 2018). In fact, exercise‐induced levels of low‐grade ischaemia induced by microvascular dysfunction might help explain several haemodynamic findings in HFpEF, such as impaired energy‐dependent relaxation or the paradoxical upward shift of the LV‐EDPVR (Rommel et al., 2016). Medical interventions for microvascular function have not shown clinical benefit in HFpEF (Redfield et al., 2015). An interventional approach, increasing coronary venous pressure through dilatation of a balloon in the coronary sinus, has recently been suggested to improve microvascular function (Ullrich et al., 2023). Whether this approach could be beneficial specifically in patients with HFpEF remains to be demonstrated.

The multifaceted reasons for an inadequate COR in HFpEF nicely illustrate that this syndrome is not confined to the heart itself but is a systemic disease. In fact, some of the proposed mechanisms (e.g., sympathetic overactivity) do not necessarily require cardiac damage as a prerequisite. Instead, dysregulation of neuro‐cardiovascular factors that regulate intracellular metabolic homeostasis and blood pressure might significantly contribute to impaired cardiac output. Importantly, this dysregulation has also been suggested to be present in healthy individuals, questioning the central and exclusive role of cardiac output in governing the regulation of cardiovascular function (Stöhr, 2022).

We acknowledge that this discussion neither exclusively covers potential therapies for HFpEF nor is collectively exhaustive. Instead, it provides an overview of how pathophysiology‐guided phenotyping might help identify targets to improve central haemodynamics, particularly COR. We also recognize that peripheral and pulmonary abnormalities might require equal consideration in many patients to improve symptoms and clinical outcomes in HFpEF.

## 9 CONCLUSION

Impaired COR stands as a central haemodynamic abnormality in HFpEF. Pathophysiological phenotyping, based on alterations in left ventricular structure and function, large artery stiffness and left ventricular afterload, atrial function, right ventricular function and pulmonary hypertension, tricuspid regurgitation and pericardial restraint, as well as chronotropic competence, might reveal specific pathologies underlying impaired stroke volume increase and suggest potential treatment options.

## AUTHOR CONTRIBUTIONS

Karl‐Philipp Rommel conceived and designed the current manuscript and Paula Sagmeister drafted the manuscript. All authors have contributed indispensable preliminary work to the substantive foundation of this review paper, interpreted the data and revised the manuscript critically. All authors have read and approved the final version of the manuscript and agree to be accountable for all aspects of the work in ensuring that questions related to the accuracy or integrity of any part of the work are appropriately investigated and resolved. All persons designated as authors qualify for authorship, and all those who qualify for authorship are listed.

## CONFLICT OF INTEREST

The authors report no conflict of interest relevant to the content of this paper.
